# Retraction: Microbiome restoration diet improves digestion, cognition and physical and emotional wellbeing

**DOI:** 10.1371/journal.pone.0194851

**Published:** 2018-03-20

**Authors:** The PLOS ONE Editors

After publication of this article [1], concerns were raised about the validity of the study design and conclusions reported. Following a post-publication assessment involving staff editors and members of our Editorial Board, the *PLOS ONE* Editors decided to retract this article due to methodological concerns that call into question the validity of the study and its conclusions which the peer review process did not adequately address.

The concerns include:

- There was no control group for this study.
- The participants were not blind to the treatment and were selected from a population for which placebo effects are anticipated. Furthermore, outcome data consist of self-reported measures including weight and symptoms. Self-reported weight data were not validated by the researchers, and no dietary measures were included to assess adherence to the diet. The validity and reliability of the tool used to assess participants' symptoms has not been demonstrated, and as no validated measures or scales were used to assess cognitive function or emotional well-being, the conclusions are not well-supported.
- The authors did not report on potential confounding variables such as medication or dietary supplement use by participants before or during the study.
- The prebiotic and probiotic components of the diet are not reported in sufficient detail to enable reproducibility. While described in general terms, quantities consumed per day and the range of such items consumed per participant are not reported.
- The authors did not report a power calculation to support the sample size and demonstrate it as adequate to assess the anticipated effects.
- Further concerns were raised about the statistical analysis methods and reporting: the authors did not report a statistical analysis plan and methodology clearly in their Methods section, and the application of Cronbach's alpha in the study has been questioned in light of the diverse symptoms assessed with the scale.
- Reference to the intervention as a "microbiome restoration" diet is not supported, as the authors did not assess microbiome composition.

In light of the above concerns about the study design and methods used, the *PLOS ONE* Editors consider that the conclusions of this study are not supported by the data presented and retract this article.

KL and JH do not agree with the retraction and stand by the article’s conclusion.
